# Second Childhood

**Published:** 1877-05

**Authors:** 


### SECOND CHILDHOOD.

THE three great necessities of infancy and childhood are: warmth, food, and exercise. As to food, milk is Nature's preparation, and is the only article known which has in it all the necessary elements of nutrition. But when we get older, the wants of the system are changed; the conditions and capabilities of the stomach are changed, and the appetite loudly calls for something more substantial. But with the weakness and years of great age, the wants of infancy return—plenty of milk, plenty of flannel; but the need of rest replaces that of ceaseless activity, and the poor old body finds its happiness in huddling over the fire all day long.

